# Effectiveness of Mild to Moderate Hypothermic Cardiopulmonary Bypass on Early Clinical Outcomes

**DOI:** 10.3390/jcdd9050151

**Published:** 2022-05-09

**Authors:** Adnan Haider, Irfan Azmatullah Khwaja, Abdul Basit Qureshi, Imran Khan, Khalid Abdul Majeed, Muhammad Shahbaz Yousaf, Hafsa Zaneb, Abdul Rehman, Imtiaz Rabbani, Sajid Khan Tahir, Habib Rehman

**Affiliations:** 1 Department of Physiology, University of Veterinary and Animal Sciences, Lahore 54000, Pakistan; adnanhaiderlecturer@kemu.edu.pk (A.H.); khalid.majeed@uvas.edu.pk (K.A.M.); drmshahbaz@uvas.edu.pk (M.S.Y.); imtiaz.rabbani@uvas.edu.pk (I.R.); sajid.tahir@uvas.edu.pk (S.K.T.); 2 Department of Cardiovascular Surgery, King Edward Medical University, Lahore 54000, Pakistan; irfan_a_khwaja@hotmail.com; 3 Department of Surgery, Services Institute of Medical Sciences, Lahore 54810, Pakistan; basitq@hotmail.com; 4 Department of Cardiolothoracic and Vascular Surgery, Almana General Hospital, AL Khobar 31952, Saudi Arabia; cardiac.imran@gmail.com; 5 Department of Anatomy and Histology, University of Veterinary and Animal Sciences, Lahore 54000, Pakistan; hafsa.zaneb@uvas.edu.pk; 6 Department of Epidemiology and Public Health, University of Veterinary and Animal Sciences, Lahore 54000, Pakistan; abdul.rehman@uvas.edu.pk

**Keywords:** cardiac surgery, inotropic support, in-hospital stay, blood loss, thrombocytopenia

## Abstract

Background: Intraoperative hypothermia is an integral part of cardiopulmonary bypass (CPB), and a precise degree of hypothermia may improve the early clinical outcomes of cardiac surgery. Presently, there is no agreement on an accurate, advantageous temperature range for routine use in CPB. To address this issue, we conducted a retrospective observational study to compare the effects of different hypothermic temperature ranges on primary (inotropic support, blood loss, and platelet count) and secondary (ventilation support and in-hospital stay) outcomes in patients undergoing elective cardiac surgery. Methods: Data were retrieved from the medical database of the Cardiovascular Surgery Department, King Edward Medical University, Lahore-Pakistan (a tertiary care hospital), dating from February 2015 to December 2017. Patients were divided into mild (34 °C to 36 °C), intermediate (31 °C to 33 °C), or moderate (28 °C to 30 °C) hypothermic groups. Results: Out of 275 patients, 245 (89.09%) fit the inclusion criteria. The cohort with mild hypothermic CPB temperatures presented better clinical outcomes in terms of requiring less inotropic support, less blood loss, fewer blood transfusions, improved platelet counts, shorter in-hospital stays, and required less ventilation support, when compared with other hypothermic groups. Conclusions: Mild hypothermic CPB (34 °C to 36 °C) may produce better clinical outcomes for cardiac surgery and improve the quality of health of cardiac patients.

## 1. Introduction

Intraoperative hypothermia during cardiopulmonary bypass (CPB) is an essential component and may have a significant impact on patient outcomes after cardiac surgery [1]. Hypothermia is desired to prevent vital organs from ischemic injury by decreasing the metabolic rate and reducing oxygen consumption [2]. Established evidence suggests that even mild hypothermia (34–35 °C) has been found to decrease the metabolic rate, resulting in lower oxygen consumption, lower production of carbon dioxide, and less use of anesthetic agents [3,4]. Hypothermic temperatures (32–34 °C) are preferred for maintaining the hemoglobin dissociation curve within the normal range by allowing for better oxygen delivery to tissues, less use of inotropic support, and early extubation [5,6]. However, hypothermia during CPB has been linked to various drawbacks, such as prolonged recovery from anesthesia, cardiac morbidity, and coagulopathy [7,8]. A meta-analysis on intraoperative hypothermia in CPB explained that even a decrease of 1 °C increased the blood loss and relative risk of transfusion [9]. Hypothermic CPB, at a temperature range of 25 °C to 32 °C, has also been associated with an impairment in coagulation related to reversible platelet dysfunction and the inhibition of activated clotting factors [10]. 

In randomized trials, there were no differences in the use of blood products, extubation time, length of in-hospital stay, the incidence of myocardial infarction, or mortality in patients who were maintained at an intraoperative temperature around 34 °C when compared with the patients who did not have hypothermia induced during CPB [11,12,13]. A recent study concluded that there were no differences in the variables of in-hospital stay, mortality, and ventilation support, however, shorter ICU stays and fewer blood transfusions were observed for patients maintained with an intraoperative temperature of 32–35 °C, compared to a temperature of >35 °C during CPB [12]. On the other hand, hypothermic CPB temperatures of >34 °C versus ≤34 °C have shown no differences in terms of mortality, need for blood transfusion, and postoperative strokes [14]. Similarly, no differences were observed in a pediatric population when comparing the impact of moderate (24 °C) and mild (34 °C) hypothermia during CPB on systemic inflammatory response and organ injury [15]. In this context, it appears that the outcomes of different hypothermic temperature ranges are unclear and inconsistent [12,16]. From the aforementioned discussion, it appears that there is a huge variation in the intraoperative hypothermic temperatures. Therefore, there is a need to define an appropriate intraoperative temperature range that may be more suitable for producing better clinical outcomes in patients undergoing CPB. Considering this reason, we retrospectively collected and analyzed data from a tertiary care hospital and compared the effects of different intraoperative hypothermic temperatures, ranging from 28 °C to 36 °C, on primary outcomes (inotropic support, blood loss, and platelet count) and secondary outcomes (ventilation requirements and in-hospital stay) in adult patients undergoing elective cardiac surgery.

## 2. Materials and Methods

### 2.1. Study Design and Patients

This is a single-center, retrospective observational study, for which data were retrieved from the registry of the Department of Cardiovascular Surgery of the King Edward Medical University/Mayo Hospital (the largest tertiary care hospital in Punjab Province) in Lahore, Pakistan. The medical records of all patients who underwent cardiac intervention between February 2015 and December 2017 were reviewed. This study was approved by the Institutional Review Board of the King Edward Medical University, Lahore (No. 501/RC/KEMU). Patients who had severe pulmonary hypertension, had preoperative uncontrolled diabetes, were on hemodialysis, had poor left ventricular function (effective ejection fraction <30%), had redo coronary artery bypass grafting, were preoperatively on an intra-aortic balloon pump, and those who were brought to some emergency or who had any ongoing infection, were excluded from the study. A genuine attempt was made to create study cohorts that were as homogeneous as possible. The patients were divided into three different groups, namely, moderate hypothermia (28 °C to 30 °C), intermediate hypothermia (31 °C to 33 °C), and mild hypothermia (34 °C to 36 °C). The authors did not interfere in any of the surgical interventions and only retrieved the data from the institute’s database. 

### 2.2. Basal Characteristics of Patients

Baseline information of the patients, such as demographic data, blood parameters, serum glutamic pyruvic transaminase (SGPT), serum glutamic oxaloacetic transaminase (SGOT), serum bilirubin, blood urea, and left ventricular ejection fraction, were extracted from the medical records. The definitions used for the study are from the Society of Thoracic Surgeons National Cardiac Surgery Database [17].

### 2.3. Intraoperative and Postoperative Data

All of the cardiac surgeries were performed under general anesthesia using a standard technique, as previously described [18]. Intraoperative variables such as CPB time, aortic cross-clamp time (ACC), blood hemoglobin concentration, urine output, and cerebral oxygen expenditure (CeO_2_) were retrieved from the medical database. The CeO_2_, calculated as a difference between arterial and jugular oxygen saturation, was noted 30 min after the initiation of CPB and, thereafter, during the rewarming phase at a temperature of 36 °C. The postoperative variables included in this study were activated clotting time (ACT), hemoglobin concentration, liver function markers (SGPT and SGOT), ICU stay, leukocyte count, platelet count, and mortality.

### 2.4. Study Endpoints

The primary endpoints of this study were inotropic support, blood loss, and platelet count, while the secondary outcomes were the duration of mechanical ventilation and in-hospital stay. The duration of mechanical ventilation was defined as the time from weaning off CPB until extubation in the ICU.

### 2.5. Statistical Analysis

The normal distribution of the data was determined using the Kolmogorov–Smirnov test. Normally distributed quantitative variables were presented as the mean ± SD, while medians and ranges were used for non-normal variables. Qualitative variables were presented as frequency and percentage. Baseline characteristics of the patients in three treatment groups (mild, intermediate, and moderate hypothermia) were compared using a one-way analysis of variance (for normally distributed variables, i.e., body mass index) Kruskal–Wallis H test (for non-normal variables). Categorical variables were compared using Pearson’s chi-squared test. To test whether the three treatments significantly predicted the primary and secondary outcomes, generalized linear models (GLMs) with different “link” functions were constructed. The effect of the three treatments on the in-hospital stay (secondary outcome) and platelet count of the patients (primary outcome) was assessed using the Poisson regression model. The association of the three treatments with blood transfusion (yes/no), the primary outcome, was modeled using logistic regression. To measure the effects of the three treatments on ventilation support (secondary outcome), blood loss, dopamine level, and adrenalin concentration (primary outcomes), quasi-Poisson regression (that accounts for overdispersion in data) was used. Gender, age, diabetes history, smoking, and BMI were assessed as potential confounding variables in all the models. A probability value less than 0.05 was considered statistically significant. The descriptive statistics were performed using the Statistical Package for the Social Sciences program (SPSS Version 26.0. Armonk, NY, USA), however, the regression models were built in R version 4.1.1 (https://www.R-project.org/) using RStudio version 2021.09.0 as an interface (http://www.rstudio.com/) assessed on 12 April 2022.

## 3. Results

Out of 275 patients, 264 (96.00%) were operated on for elective cardiac surgery. The remaining 11 (4.00%) were operated on as emergency cases. However, 245 (89.09%) patients fit the study inclusion criteria. The number of patients included in the moderate hypothermic (28 °C to 30 °C), intermediate hypothermic (31 °C to 33 °C), and mild hypothermic (34 °C to 36 °C) groups were 81, 89, and 75, respectively. The distribution of surgical procedures among the three groups is presented in Table 1.

As shown in Table 2, the groups were homogeneous in terms of age, body weight, body mass index, gender distribution, history of diabetes mellitus, smoking, ejection fraction, EuroSCORE-II, serum concentrations of blood urea, SGPT, SGOT, white blood cell count, and platelet count.

Table 3 shows the results of multivariable regression analysis which indicated that more (*p* < 0.003) inotropic support, in terms of adrenaline infusion rate, was required in the moderate hypothermic group compared to the mild hypothermic group. Similarly, a higher dopamine infusion rate was required for the intermediate group compared to the mild hypothermic group. Blood loss was significantly higher (*p* < 0.001) in intermediate and moderate hypothermic groups; incidence rate ratios (IRR) = 1.75 (1.58–1.95) and 1.73 (1.56–1.93), respectively. The odds of requiring a blood transfusion in patients in the intermediate and moderate hypothermic groups were four (OR, 4.50; 95% CI, 1.59–16.17; *p =* 0.009) and five times (OR, 5.82; 95% CI, 2.07–20.84; *p =* 0.002) higher, respectively, compared to the mild hypothermic group. Consequently, platelet count was lower (*p* < 0.001) in both the intermediate and moderate hypothermic groups. For secondary endpoints, in-hospital stay was significantly higher in the intermediate (IRR = 1.12; *p* = 0.046) and moderate hypothermic groups (IRR = 1.13; *p* = 0.034) compared to the mild hypothermic group. Greater ventilation support was required for patients maintained at intermediate (IRR = 1.27; *p* = 0.01) and moderate (IRR = 1.80; *p* < 0.001) hypothermia.

Intraoperative and postoperative parameters showed that ACC time was shorter (*p* < 0.05) in the mild hypothermic group compared with the other groups (Table 4). During the intraoperative phase, CeO_2_ after thirty minutes of initiation of CPB, as well as during the re-warming phase, were higher (*p* < 0.01) in the mild hypothermic group compared with the other groups (Table 4). The intraoperative temperature did not influence CPB time, hemoglobin concentration, blood sugar, leukocyte count, ICU stay, and mortality among the groups (Table 4).

## 4. Discussion

In recent decades, there has been considerable debate regarding the optimization of intraoperative temperature in CPB [12,19,20]. Nevertheless, it is still believed that hypothermic CPB is an effective strategy for protecting the functionality of visceral organs during cardiac surgery, resulting in better organ perfusion, as well as a reduction in bypass flow [21]. Therefore, our institute follows the mild to moderate intraoperative hypothermic management of patients in CPB. For improved clinical outcomes in CPB, it has become imperative to identify an appropriate temperature range. Consequently, we fixed a temperature difference of 3 °C during CPB, defined as moderate hypothermia (28 °C to 30 °C), intermediate hypothermia (31 °C to 33 °C), and mild hypothermia (34 °C to 36 °C). This study suggests that mild hypothermic CPB appears safer and has more early clinical advantages compared with the other groups in adult cardiac surgery. In the current study, the significant differences for primary endpoints, translated in the favor of the mild hypothermic group, were less inotropic support, reduced blood loss, and improved platelet count. Shorter extubation time and a shortened in-hospital stay were also found in the mild hypothermic group as secondary outcomes.

The inotropic support at the end of cardiac surgery may indicate morbidity and mortality in adult patients [22]. Taking inotropic support as a surrogate parameter for intraoperative ischemia, this study demonstrated that the lower temperature required more and longer inotropic support than the mild hypothermic group. Similar results have also been reported, describing that the patients with higher temperatures needed less inotropic support compared with those managed at relatively lower hypothermic temperatures during CPB [6,23,24]. In another study, Stocker and his colleagues revealed that inotropic support tended to be higher in children with a 24 °C CPB temperature compared with a 34 °C group [15]. The longer duration of inotropic support in patients with a very low CPB intraoperative temperature may be due to multiple factors, such as microcirculatory dysfunction, capillary leakage, endotoxin release, reduced parenchymal oxygen supply, and lower myocardial contractility [25]. 

Currently, our mild hypothermic group cohort required few transfusions, as blood loss was low compared with the other hypothermic groups (Table 3). A meta-analysis involving a cohort of 2000 patients who underwent operations for different surgeries (including cardiothoracic), suggested that even mild hypothermia increased postoperative bleeding and consequently enhanced the need for blood transfusions [9]. The effects of hypothermia on platelet function are not fully understood and have been inconsistently reported in the published literature. Some studies have shown that hypothermia changes platelet morphology and increases platelet margination and splenic sequestration, causing thrombocytopenia [26]. However, hypothermia also appears to enhance shear-induced platelet aggregation and prolongs retention of the von Willebrand factor on the platelet’s surface [27,28]. The net hemostatic effects from these changes may also depend on the degree and duration of hypothermia. Hypothermia also reduces procoagulant enzyme activity and increases fibrinolysis, which could contribute to postoperative bleeding [29,30,31].

In our CPB temperature management protocol, we found that the influence of hypothermia-induced coagulopathy was not pronounced in the mild hypothermic group compared with other hypothermic CPB patients, as the number of platelets was greater in the former group. Hypothermia has been found to induce thrombocytopenia, inhibit the formation of platelets plugs, or alter the fibrinolytic cascade system [32]. These factors may lead to blood loss and, consequently, a greater need for blood transfusions [33,34,35]. We also observed less blood loss in the mild hypothermic group compared with the other groups. Consistent with our study, Mahla and his fellow researchers also identified more platelets in the mild temperature range compared with lower temperatures [36].

The temperature management protocol during CPB also translated into secondary endpoints. We found, in agreement with a previous study, that patients, who were operated on at a higher hypothermic CPB temperature range, had shorter in-hospital stays compared with the patients of lower temperature groups [24]. Morbidity can be defined as the length of in-hospital stays with significant complications, and also includes patients who died [37]. Therefore, the length of an in-hospital stay is a marker of morbidity and is influenced by other factors, such as individual and institutional practices [38]. Hence, with shorter in-hospital stays, there is reduced strain due to the waiting list of patients and the associated costs. 

Likewise, we also found that approximately double the duration of mechanical support was required for patients in the moderate hypothermia group compared with the mild hypothermia group (Incidence Rate Ratio (95% CI) = 1.80 (1.51–2.14); *p* < 0.001). A body of evidence has demonstrated that a longer duration of mechanical ventilation is required for patients maintained on hypothermic CPB of <26 °C, suggesting a temperature-induced dysfunction of vessels in the microcirculation [6,24,39]. Others presumed that longer ventilation support after CPB may be due to a temperature-dependent immunological response of lungs that are very sensitive to CPB [40,41].

Better organ protection remains a challenge for successful CPB. Intraoperative hypothermia during CPB reduces tissue metabolism. Most studies only highlighted the relative changes in CeO_2_ as a measure of the cerebral demand for oxygen during cardiac and non-cardiac surgeries [42]. We reported these changes on a two-time scale. We found that CeO_2_ levels decreased in the hypothermic CPB groups after 30 min of CPB, and this fall in oxygen demand was proportionate to the CPB temperature range. Experimental results also support these findings, wherein CeO_2_ was found to decrease during the lower hypothermic CPB temperature range and increased during the higher hypothermic CPB range [5,43]. The findings of this study will help support clinical decisions through the selection of the most appropriate hypothermic condition for better clinical outcomes.

## 5. Conclusions

Our study demonstrates that mild hypothermia is better than intermediate or moderate hypothermia during CPB in terms of lower inotropic support, less blood loss, improved platelet count, shorter in-hospital stay, and less ventilation support.

## 6. Limitations

The results of the present study should be carefully interpreted because of the following limitations. First, the allocation of patients to the various treatment groups was purely based on the surgeon’s decision, which might have resulted in assigning the more stable patients to the mild hypothermia group, leading to somewhat inflated incidence rate ratios for the intermediate and moderate groups. Since it was a retrospective study, the researcher had no control over the data recording. However, setting a research question retrospectively (but before carrying out analyses) could be beneficial because it could decrease information bias in the data recording [44]. Although a multivariable analysis was performed to control for confounding effects, unmeasured confounders might have introduced a potential bias. Secondly, the variables were only recorded during the in-hospital stay of the patients. Therefore, the effects of these hypothermic temperature ranges cannot be applied to long-term clinical outcomes. Finally, the majority (72.65%) of patients were treated for CABG, followed by mitral valve replacement (13.06%). For this reason, the findings of the present study cannot be generalized to other cardiac surgical interventions. However, the strength of this study is the exclusion of bias related to surgical teams as the same surgeon and perfusionist were involved in the CPB procedure.

## Figures and Tables

**Table 1:** Distribution of surgical procedures in hypothermic groups.

Surgical Procedure	Type of Hypothermia			Total *n* (%)	*p*-Value
	Moderate *n* (%)	Intermediate *n* (%)	Mild *n* (%)		
Atrial septal defect	1 (1.23)	1 (1.12)	1 (1.33)	3 (1.22)	0.993
Aortic valve replacement	2 (2.46)	9 (10.11)	4 (5.33)	15 (6.12)	0.109
Coronary artery bypass grafting (CABG)	62(76.54)	60 (67.41)	56 (74.66)	178 (72.65)	0.368
CABG (1 graft)	12 (19.35)	08 (13.33)	11 (19.64)	31 (17.41)	0.428
CABG (2 grafts)	20 (32.25)	29 (48.33)	28 (50.00)	77 (43.25)	0.164
CABG (3 grafts)	30 (48.38)	23 (38.33)	18 (32.14)	71 (39.88)	0.214
CABG + Mitral valve replacement	0 (0.00)	2 (2.24)	0 (0.00)	2 (0.81)	0.171
CABG + Aortic valve replacement	1 (1.23)	1 (1.12)	0 (0.00)	2 (0.81)	0.639
Double valve replacement	3 (3.70)	1 (1.12)	4 (5.33)	8 (3.26)	0.308
Mitral valve replacement	11 (13.58)	13 (14.60)	8 (10.66)	32 (13.06)	0.746
Triple valve replacement	1 (1.23)	2 (2.24)	2 (2.66)	5 (2.04)	0.807
Total	81 (33.07)	89 (36.32)	75 (30.61)	245 (100.00)	0.581

Moderate hypothermia (28 °C to 30 °C); intermediate hypothermia (31 °C to 33 °C); mild hypothermia (34 °C to 36 °C). Data are presented as *n* = number of patients and percentage, with Pearson’s chi-squared test to calculate the *p*-value.

**Table 2:** Baseline characteristics of patients undergoing cardiopulmonary bypass.

Parameter	Types of Hypothermia; Median (Range)			*p*-Value
	Moderate (*n* = 81)	Intermediate (*n* = 89)	Mild (*n* = 75)	
Age (years)	55 (24–75)	54 (19–70)	55 (20–73)	0.777
Gender; male; *n* (%) ^†^	66 (81.00)	73 (82.02)	58 (77.33)	0.720
History of diabetes mellitus; *n* (%)	32 (39.50)	26 (29.21)	21 (28.00%)	0.275
History of smoking; *n* (%)	20 (24.69)	23 (25.84)	17 (22.66)	0.894
Weight (kg)	71.10 (51.90–126.10)	71.70 (50.80– 127.40)	72.40 (50.90–96.50)	0.216
Height (cm)	165 (143–190)	169 (146–195)	164 (140–193)	0.599
Body mass index (kg/m^2^) ^‡^	27.12 ± 4.30	26.60 ± 5.00	26.06 ± 4.35	0.361
Ejection fraction (%)	55 (30–57)	52 (32–57)	57 (30–58)	0.444
EuroSCORE-II	1.40 (1.10–3.60)	1.50 (1.00–4.00)	1.40 (1.20–3.80)	0.870
Blood urea (mg/dL)	32 (16–83)	30 (11–73)	29 (17–56)	0.621
Bilirubin (mg/dL)	0.70 (0.30–2.60)	0.60 (0.20–2.00)	0.80 (0.40–2.40)	0.040
SGPT (U/L)	28 (12–181)	29 (7–121)	25 (15–156)	0.559
SGOT (U/L)	35 (13–297)	34 (12–159)	35 (16–151)	0.412
Leukocyte count (10^3^/μL)	9.25 (4.90–19.50)	9.37 (4.20–19.60)	9.50 (5.20–19.80)	0.575
Platelets count (10^3^/μL)	202 (68–662)	242 (87–661)	235 (130–591)	0.182

Moderate hypothermia (28 °C to 30 °C); intermediate hypothermia (31 °C to 33 °C); mild hypothermia (34 °C to 36 °C). ^†^ Data are presented as *n* = number of patients and percentage. ^‡^ Values are expressed as mean ± SD. SGPT = Serum glutamic pyruvic transaminase; SGOT = Serum glutamic oxaloacetic transaminase.

**Table 3:** Multivariable regression analysis for primary and secondary endpoints.

	Reference Group *	Incidence Rate Ratios/Odds Ratio (95% CI)		R^2^ Nagelkerke
	Mild	Intermediate	Moderate	
Primary endpoints:				
Adrenaline infusion rate *p*-value	0.06 (0.05–0.06) mcg/kg/min -	0.96 (0.81–1.14) 0.652	1.29 (1.09–1.52) 0.003	0.066
Dopamine infusion rate *p*-value	3.88 (3.70–4.06) mcg/kg/min -	1.09 (1.02–1.16) 0.006	1.00 (0.94–1.07) 0.944	0.05
Total Blood loss *p*-value	383.87 (352.09–417.50) mL -	1.75 (1.58–1.95) <0.001	1.73 (1.56–1.93) <0.001	1.00
Blood transfusion ** p-value	Reference -	4.50 (1.59–16.17) 0.009	5.82 (2.07–20.84) 0.002	0.046
Platelet count *p*-value	270.4 (265.0–275.9) × 10^3^/μL -	0.95 (0.93–0.97) <0.001	0.88 (0.86–0.90) <0.001	0.82
Secondary endpoints:				
Ventilation time *p*-value	126.8 (109.8–145.4) minutes -	1.27 (1.06–1.52) 0.010	1.80 (1.51–2.14) <0.001	1.00
In-hospital stay *p*-value	7.05 (6.47–7.67) days -	1.12 (1.00–1.26) 0.046	1.13 (1.01–1.27) 0.034	0.108

* Baseline values of the endpoints for the reference group, i.e., mild hypothermia, considering a reference IRR = 1.00. ** The values for blood transfusion are presented as odds ratios (95% CI) and R^2^ *Tjur.* Moderate hypothermia (28 °C to 30 °C); intermediate hypothermia (31 °C to 33 °C); mild hypothermia (34 °C to 36 °C).

**Table 4:** Intraoperative and postoperative characteristics of patients undergoing cardiopulmonary bypass (CPB).

Parameter	Types of Hypothermia; Median (Range)			*p*-Value
	Moderate (*n* = 81)	Intermediate (*n* = 89)	Mild (*n* = 75)	
Intraoperative Phase:				
CPB time (minutes)	106 (61–264)	110 (52–225)	100 (57–191)	0.058
ACC (minutes)	68 (29–148)	72 (28–169)	55 (34–171)	0.009
Blood sugar random (mg/dL) ^†^	207.30 ± 71.03	224.21 ± 65.70	218.11 ± 70.03	0.274
CeO_2_ at 30 min (%)	23 (20–25)	24 (20–27)	32 (30–34)	<0.001
CeO_2_ at rewarming (%)	35 (26–40)	32 (28–35)	34 (28–37)	<0.001
Hemoglobin (g/dL) ^†^	8.61 ± 1.39	8.79 ± 1.78	8.90 ± 1.74	0.540
Postoperative Phase:				
Hemoglobin (g/dL) ^†^	9.80 ± 1.462	9.76 ± 1.546	9.93 ± 1.467	0.761
SGPT (U/L)	31 (14–617)	33 (15–256)	34 (11–359)	0.728
SGOT (U/L)	62 (19–310)	61 (22–327)	51 (21–384)	0.047
Leukocyte count (10^3^/μL)	18.90 (7.80–48.00)	17.90 (5.90–30.70)	18.70 (9.30–48.80)	0.422
ICU stay (days)	5 (3–10)	5 (1–9)	5 (1–10)	0.636
Mortality; *n* (%) ^‡^	1 (1.23)	1 (1.12)	0 (0.00)	0.639

Moderate hypothermia (28 °C to 30 °C); intermediate hypothermia (31 °C to 33 °C); mild hypothermia (34 °C to 36 °C); ACC = Aortic cross-clamp time; CeO_2_ = Cerebral oxygen expenditure; SGPT = Serum glutamic pyruvic transaminase; SGOT = Serum glutamic oxaloacetic transaminase. ^†^ Data are presented as *n* = number of patients and percentage. ^‡^ Values are expressed as mean ± SD.

## Data Availability

The data presented in this study are available upon request from the corresponding author and are not publicly available due to ethical issues.
